# Color calibration and fusion of lens-free and mobile-phone microscopy images for high-resolution and accurate color reproduction

**DOI:** 10.1038/srep27811

**Published:** 2016-06-10

**Authors:** Yibo Zhang, Yichen Wu, Yun Zhang, Aydogan Ozcan

**Affiliations:** 1 Electrical Engineering Department, University of California, Los Angeles, CA, 90095, USA; 2 Bioengineering Department, University of California, Los Angeles, CA, 90095, USA; 3 California NanoSystems Institute (CNSI), University of California, Los Angeles, CA, 90095, USA; 4 Department of Surgery, David Geffen School of Medicine, University of California, Los Angeles, CA, 90095, USA

## Abstract

Lens-free holographic microscopy can achieve wide-field imaging in a cost-effective and field-portable setup, making it a promising technique for point-of-care and telepathology applications. However, due to relatively narrow-band sources used in holographic microscopy, conventional colorization methods that use images reconstructed at discrete wavelengths, corresponding to e.g., red (R), green (G) and blue (B) channels, are subject to color artifacts. Furthermore, these existing RGB colorization methods do not match the chromatic perception of human vision. Here we present a high-color-fidelity and high-resolution imaging method, termed “digital color fusion microscopy” (DCFM), which fuses a holographic image acquired at a single wavelength with a color-calibrated image taken by a low-magnification lens-based microscope using a wavelet transform-based colorization method. We demonstrate accurate color reproduction of DCFM by imaging stained tissue sections. In particular we show that a lens-free holographic microscope in combination with a cost-effective mobile-phone-based microscope can generate color images of specimens, performing very close to a high numerical-aperture (NA) benchtop microscope that is corrected for color distortions and chromatic aberrations, also matching the chromatic response of human vision. This method can be useful for wide-field imaging needs in telepathology applications and in resource-limited settings, where whole-slide scanning microscopy systems are not available.

Lens-free on-chip microscopy that is based on digital in-line holography emerged as a promising technique for point-of-care imaging and pathology applications, with the advantages of significantly larger field of view (FOV) than conventional lens-based microscopy tools, light weight, compactness and field portability^1^,^2^,^3^,^4^,^5^,^6^,^7^,^8^,^9^,^10^,^11^. In a lens-free imaging setup (Fig. 1, upper-left), a partially-coherent light source that is butt-coupled to an optical fiber or filtered by a pinhole is used to illuminate the sample, and a complementary metal-oxide semiconductor (CMOS) or charge-coupled device (CCD) image sensor is placed under the sample to record its diffraction pattern (i.e., an in-line hologram of the sample). In this imaging set-up, the source-to-sample distance (typically, *z*_*1*_ ~ 5–15 cm) is much larger than the sample-to-image-sensor distance (typically, *z*_*2*_ < 1 mm), and therefore the imaging FOV is equal to the active area of the image sensor chip (~20.5 mm^2^). The recorded hologram is then digitally processed by iterative phase retrieval algorithms to reconstruct amplitude and phase images of the object^1^,^2^,^3^,^5^,^6^,^12^,^13^,^14^,^15^,^16^,^17^,^18^. This simple optical design makes it possible for the lens-free imaging device to be extremely cost-effective, compact, and field-portable. Additional hardware and software can be incorporated into the same design to achieve e.g., higher resolution, better image quality and differential contrast imaging^4^,^19^,^20^. For example, pixel super-resolution (PSR) methods^5^,^7^,^11^,^21^,^22^,^23^ can be applied to break the resolution limit imposed by the pixel size of the image sensor chip, and multi-height-based phase recovery techniques^3^,^6^,^7^,^24^ can be used to remove twin-image related artifacts especially when imaging dense and connected samples such as tissue sections used in pathology.

The holographic imaging principle of lens-free on-chip microscopy inherently limits the illumination to narrow-band sources. Thus, to obtain a color image, reconstructions at multiple wavelengths are usually needed. For example, some of the previously used lens-free RGB colorization methods^1^,^2^,^3^,^11^ combine the image reconstructions at three discrete wavelengths selected from the red, green and blue regions of the visible spectrum. Unfortunately these RGB-combination methods are subject to color distortions since the chromatic response of human vision, defined by the Commission internationale de l′éclairage (CIE) standard observer^25^, has rather broad responsivity curves (i.e., *color matching functions*). Therefore, a simple combination of holographic images recorded at red, green and blue illumination wavelengths, with relatively narrow peaks, is not a good representation of the chromatic response of human vision. Stated differently, combination of holographic images that are acquired at a few discrete wavelengths cannot satisfy the Luther condition, which states that observer metamerism color error is minimized when the spectral responsivities are linear combinations of the color matching functions^26^.

On the other hand, conventional lens-based incoherent bright-field microscopy has a natural advantage in color reproduction since broadband or “white” light sources are used for illumination, and the spectral sensitivity curves of the color filter arrays (CFA) employed in digital image sensor chips (CMOS or CCD) are designed to approximate the color matching functions of the human visual system^27^. Furthermore, a variety of color calibration methods exist to further improve the color fidelity of bright-field microscopy systems^28^,^29^,^30^,^31^,^32^,^33^,^34^,^35^.

Here, we propose a method to bridge the advantages of lens-free microscopy with those of lens-based microscopes to achieve high color accuracy, high resolution and wide FOV at the same time (Fig. 1). This method uses a lens-free holographic microscope using a single wavelength of illumination and a low-magnification lens-based incoherent bright-field microscope (e.g., a mobile-phone based microscope) to generate color corrected wide-field and high-resolution images of specimen. We term this technique as “digital color fusion microscopy” (DCFM). For optimal color performance, lens-based microscope images are digitally color-calibrated using a polynomial regression based calibration framework^28^ and denoised^36^. The grayscale holographic image of the lens-free microscope and the lens-based bright-field color image are then merged using a discrete wavelet transform based algorithm to generate the final color image of the specimen, where the high-resolution components (spatial details) come from the lens-free image and the low-resolution components come from the lens-based color image^37^. Because our lens-based imaging system employs low-magnification and low-NA, its FOV can be matched to the FOV of the lens-free microscope by digitally tiling together a few lens-based images. This novel color fusion approach combines a holographic microscope and an incoherent lens-based mobile imaging device to utilize their respective advantages, while also mitigating their respective drawbacks/limitations.

We demonstrate the accuracy of DCFM by imaging stained breast cancer tissue sections and show that a lens-free holographic microscope in combination with a portable and inexpensive mobile-phone-based microscope can generate color images of specimen that are very close to the images acquired by a color-corrected high-NA benchtop microscopy system. The overall color imaging performance of DCFM surpasses previous demonstrations of lens-free microscopy and mobile-phone based microscopy. We believe that this digital colorization method can be very useful for wide-field imaging needs related to point-of-care and telepathology applications, especially in resource-scarce settings.

## Methods

### Lens-free on-chip imaging setup

A broadband source (WhiteLase micro, Fianium Ltd.) is filtered by an acousto-optic tunable filter to output partially coherent light within the visible spectrum with a bandwidth of ~2.5 nm. The light is coupled into a single-mode optical fiber with an adjusted output power of ~20 μW, which illuminates a sample that is mounted on a 3D-printed slide holder that places the sample ~5–15 cm (*z*_*1*_ distance) below the fiber tip. A CMOS image sensor chip (IMX081, Sony, 1.12 μm pixel size) is placed ~100–600 μm (*z*_*2*_ distance) below the sample and is attached to a positioning stage (MAX606, Thorlabs, Inc.) for image sensor translation to perform pixel super-resolution and multi-height based phase recovery. A LabVIEW program coordinates different components of this setup during the image acquisition, and a desktop computer (OptiPlex 9010, Dell Inc.) is used to process the image data. The detailed optical setup is illustrated in Supplementary Fig. S1.

### Design and assembly of the mobile-phone-based microscope

A custom-designed attachment module built with cost-effective optomechanical components and 3D-printed housing (3D printer: Dimension Elite, Stratasys, Ltd.) is attached to a camera phone. Within the attachment, a white LED (MLEAWT-A1-R250-0004E5, Cree Inc.) powered by three AAA batteries is placed behind a diffuser to give uniform illumination on the sample that is mounted on a custom-fabricated x-y-z translation stage. The sample is placed in close proximity to an external lens (2.6 mm focal length), while the lens is placed right next to the camera module of the mobile phone. The focusing and sample translation can be both achieved by turning the knobs of the x-y-z translation stage. The opto-mechanical design of our mobile microscope is further illustrated in Supplementary Fig. S2.

### Digital fusion of a high-resolution lens-free mono-color image with a low-resolution lens-based color image using a wavelet transform-based algorithm

**Figure 2:**
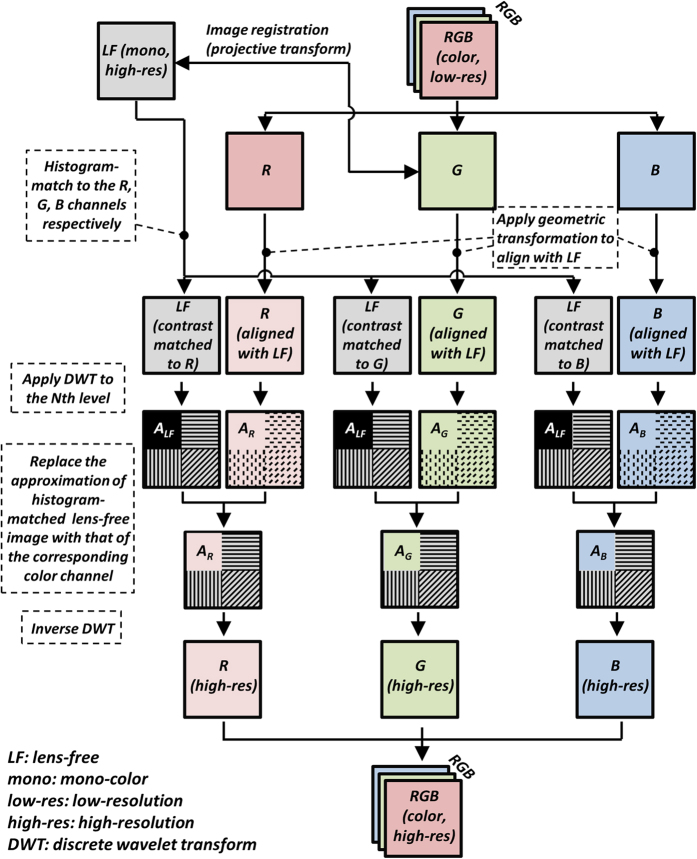
Flow chart of our wavelet-based image fusion algorithm. Refer to the Methods Section for further details.

The effective pixel sizes of the lens-free mono-color image and the lens-based color image are first matched through image resizing. Then, as shown in Fig. 2, the green channel of the RGB image is extracted to perform automated image registration against the lens-free image. Here we use automated feature matching implemented using the Computer Vision System Toolbox of MATLAB. A geometric transformation matrix is calculated based on automatic matching of spatial features, and this transformation is applied to all the R, G and B components, resulting in a registered image that is aligned with the lens-free image. After this image registration step, the contrast of the lens-free reconstructed mono-color image is matched to the R, G and B channels respectively through histogram matching^38^. Discrete wavelet transform (biorthogonal 3.7 basis^39^) is next applied to each contrast-matched lens-free image and the R, G, B channels of the lens-based image. The number of levels of the wavelet decomposition (N) mostly depends on the resolution gap between the lens-free image and the low-magnification lens-based image and in this manuscript we used N ~ 4–6 levels. In the wavelet domain, for each channel (R, G and B) we create a fused image by taking the low-resolution components (approximation) from the lens-based image and the high-resolution components (detail) from the lens-free image, and putting them into a single image. The fused images of the R, G and B channels in the wavelet domain are inverse wavelet transformed (biorthogonal 3.7 basis) to the spatial domain, and combined into a single RGB image.

### Color calibration of a bright-field lens-based microscope using polynomial regression

**Figure 3:**
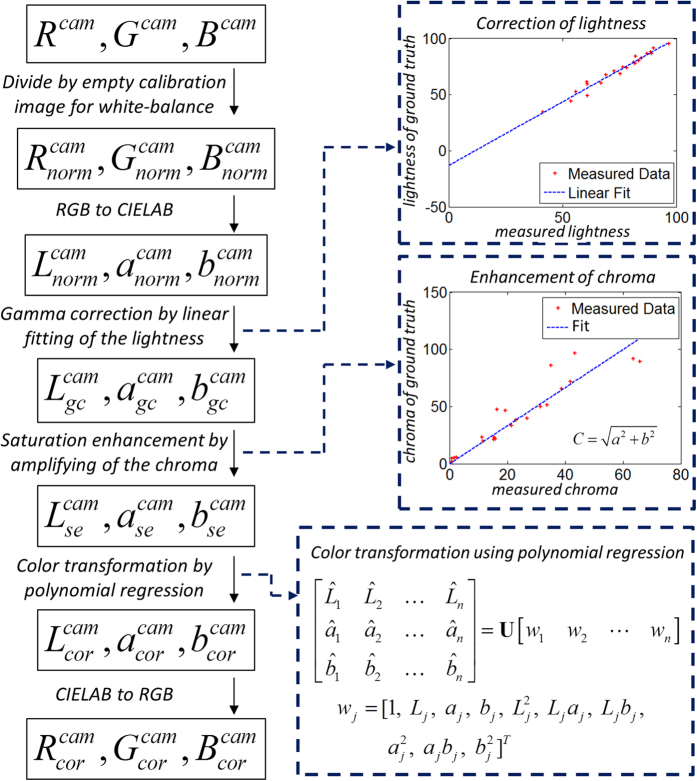
Color calibration algorithm based on polynomial regression. After this initial calibration, the color correction functions and transformations are digitally saved to be used to correct any captured image using the same calibrated imaging system.

This initial color calibration procedure needs to be conducted only once for each lens-based imaging system (i.e., for each lens/microscope combination) and it can be performed using a color checker whose actual colors (ground truth) are well known, e.g., through spectral measurements made with an optical spectrum analyzer or a wavelength scanning source. In this paper, we used lens-free hyperspectral imaging as an alternative method for color calibration. The purpose of this color calibration step is to find a mapping that corrects the colors captured by a digital microscope by re-mapping them to the actual colors defined by the ground truth. In this manuscript, we adopted a color calibration procedure that is based on polynomial regression^28^. This procedure, as outlined in Fig. 3, involves four steps: (1) image normalization and white balance, (2) correction of lightness, (3) correction of desaturation, and (4) color transformation, which will be detailed below.

#### (1) Image normalization and white balance

The captured image of the sample is normalized by an empty calibration image taken without a sample to compensate for the responsivity differences of different color-pixels and the possible deviation of the illumination source from standard illuminant (i.e., the standard spectrum of daylight, D65)^25^. Note that if the experimental parameters such as the illumination power, exposure time, filters in the light path, white-balance options of the camera, etc. are repeatable each time, this calibration process needs to be done *only once* for each lens/camera combination. The RGB image after this normalization is converted into the CIELAB color space^25^, where the *L* component represents the lightness, while *a* and *b* components represent the color or chroma. Note that the RGB color space used throughout this paper is the linear RGB color space.

#### (2) Correction of lightness

A fitting function is calculated between the lightness (*L*) component of the output of (1) and the *L* component of the ground truth. Generally a polynomial function can be used to describe the fitting, but a linear fit is also sufficient when the camera response to light intensity is linear, without a gamma correction step.

#### (3) Correction of desaturation

The saturation of the captured image is enhanced to match that of the ground truth by appropriately scaling the chroma component 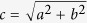. A scaling factor is calculated between the chroma of the output of (2) and the chroma of the ground truth in the least-square sense. We then use this scaling factor to modify *a* and *b* components of the output of (2).

#### (4) Color transformation

After steps (1–3), the color representation of the sample image gets closer to the actual colors, but there can still be additional sources of error, depending on the properties of the camera and the optical components. To mitigate these additional error sources, we next calculate a transformation between the *L*, *a*, *b* components of the output of (3) and the *L*, *a*, *b* components of the ground truth colors. For this purpose, a polynomial regression to the 2^nd^ order is used and a color transformation matrix **U** is calculated. Let us assume that 
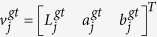
 denotes the color coordinates of the ground truth color of the *j*^th^ patch of the color checker 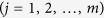, *m* is the total number of patches, and that 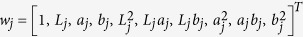
contains the elements of the 2^nd^-order polynomial in *L*_*j*_, *a*_*j*_ and *b*_*j*_ which represent the color coordinates of the same color patch captured by the camera. Our color transformation matrix **U** can be calculated by solving the following equation, in the least-square sense:


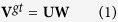


where 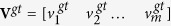 and 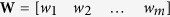.

The above detailed steps are conducted sequentially such that at the end of each step, the colors of the object image get closer to the ground truth. The color correction functions/transformations are digitally stored for each imaging system, and are used to calibrate the captured images using the same set of procedures.

### Image denoising and digital compensation for optical aberrations

Our mobile-phone based microscope uses a cost-effective image sensor chip and low-cost LED lighting, and therefore the level of noise in its images is relatively high compared to a benchtop microscope. In addition, the inexpensive external lens module that is placed in front of the mobile-phone camera results in additional optical aberrations. To mitigate these limitations, image denoising and aberration correction were digitally performed to the images obtained through our mobile-phone microscope. For this purpose, the raw image from the mobile-phone microscope was converted into raw .tif format, which was then demosaiced, resulting in an RGB image. A region of interest was cropped out from this RGB image, and the R, G, B channels were aligned to each other using feature-matching-based image registration assuming a projective transform. Then a wavelet hard-threshold denoising algorithm is performed to each channel (R, G and B) of the color image^36^. The resulting mobile-phone image is ready for subsequent color calibration and image fusion steps.

### Fabrication of transmission color checker patterns

**Figure 4:**
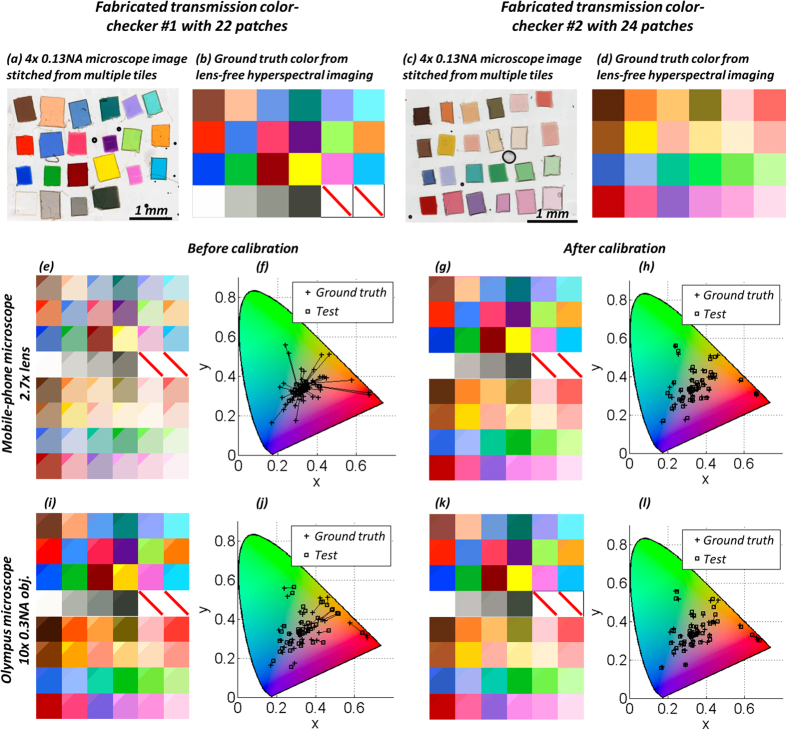
Color calibration results of a mobile-phone based microscope and a benchtop microscope. (**a**) A transmission-mode color checker (#1) with 22 patches was custom-fabricated. (**b**) The measured colors of each patch of (**a**) obtained using lens-free hyperspectral imaging, which were used as the ground truth in the training of the color calibration. (**c**) A color checker (#2) with 24 additional patches was fabricated using the same procedure as in (**a**). These additional color patches were used to improve the accuracy of the color calibration. (**d**) The ground truth colors of (**c**) measured by lens-free hyperspectral imaging. (**e**) Colors captured by our mobile-phone microscope using 2.7× magnification before the color calibration is applied. The small triangle in the upper left corner of each patch is the ground truth color from (**b**,**d**), while the rest of the patch is the color captured by the mobile-phone based microscope. (**f**) The colors in (**e**) plotted in the CIE 1931 chromaticity diagram, where “+” represents ground truth color and “” represents the captured color. Without calibration, the color aberration is quite large. (**g**,**h**) the colors after the color calibration is performed, using the same convention as (**e**,**f**). The colors after this calibration match to the ground truth very well. The quantitative color errors are also listed in Table 1. (i–l) The same process as in (**e**–**h**) using a benchtop microscope with a 10× 0.3 NA objective lens.

We custom fabricated two transmission-mode color checker patterns to calibrate the colors of our imaging systems (Fig. 4(a,c)). These two color checkers were composed of 46 patches in total, with each patch cut from one color filter from the Roscolux filter booklet. The second color checker with 24 patches was an addition to the first checker to increase the number of samples in the color space. These filters were first cut into ~0.5–1 mm squares, and were arranged onto a glass microscope slide. Next, a drop of NOA 61 (Norland Products, Inc.) was put on top of the filter patches, and a piece of glass cover slip was put on top for enclosure.

### Ground truth color calibration using hyperspectral lens-free imaging

The ground truth of the color patches (Fig. 4(b,d)) was determined using hyperspectral lens-free imaging. The lens-free microscope was used to image the color checker at wavelengths from 400 nm to 700 nm with 10 nm intervals, which is sufficient as the color matching functions are relatively broadband curves^25^. The spatial dimensions of all the color checkers can fit directly into the FOV of the lens-free microscope, thus there is no need for mechanical scanning, which is an advantage for using lens-free hyperspectral imaging to create ground truth color calibration. For each wavelength, multi-height phase recovery using 10 heights with 15 μm intervals was used. Pixel super-resolution was not implemented here due to the relatively large size of each color patch (~0.5–1 mm). The normalized hyperspectral intensity reconstructions of the patches directly reflect the transmission of the patches at different wavelengths. After extracting these transmission spectra, we correlated them with the color matching functions of human perception (CIE standard observer^25^) to get the XYZ color coordinates, from which, the RGB values can be calculated.

### Holographic image reconstruction using the angular spectrum approach

Assuming that the phase information of an optical wavefront is recovered, which will be detailed in the next sub-section, a complex hologram can be digitally back-propagated to the object plane to reveal its spatial structure using the angular spectrum approach^1^,^40^. First, the hologram is Fourier transformed to the spatial frequency domain using a fast Fourier transform to obtain its angular spectrum. Then, the angular spectrum is multiplied by a phase factor^20^, which is determined by the distance of object-to-sensor planes, wavelength and refractive index of the medium. The resulting digitally propagated spatial frequency spectrum is then inverse Fourier transformed to obtain the object’s image. The object-to-sensor distance that is used in this angular spectrum approach can be estimated using an auto-focusing algorithm^1^,^6^,^41^.

### Multi-height based iterative phase recovery

To retrieve the phase information of in-line holograms, we used a multi-height based iterative phase recovery approach^1^,^3^,^6^,^7^. During the image acquisition, the object is translated along the z direction multiple times (usually 5–10) with increments of 10–20 μm. This multi-height hologram stack is used to recover the phase information using the following algorithm.

#### Obtain an initial guess of the lost phase

We used the solution of the transport of intensity equation (TIE)^42^,^43^ as our initial phase guess. TIE is in general a lower resolution deterministic phase recovery method^42^,^43^, which is able to solve for the optical phase from multiple holograms (or diffraction patterns) acquired at different *z* positions. In our manuscript, two holograms from the multi-height hologram stack are utilized to perform the TIE based phase recovery.

#### Iterative phase recovery

By convention, we sort the acquired holograms by their vertical distance in ascending order, i.e., the closest *z* corresponds to the first hologram. After an initial phase guess that is estimated using the TIE, we start our iterative phase recovery algorithm by constructing an initial guess of the complex optical wave at the first height, whose amplitude is the square root of the first measured hologram and whose phase is the initial phase guess from from the earlier step. This complex optical wave is then digitally propagated to the second height, where its amplitude is averaged with the square root of the second hologram, and the phase is kept. The same procedure is repeated to the third height, fourth height, etc. and then backwards, with the phase being refined at each step. After typically ~10–20 iterations the algorithm converges. Besides the phase, the amplitude of each hologram plane is also refined in this process, as the averaging operation reduces the noise occurring at each captured hologram.

#### The complex wave defined by the converged amplitude and phase of a given hologram plane/height is back-propagated to the object plane

This final step can be done for any one of the hologram planes.

### Digital tilt correction using rotational field transformation

The above outlined multi-height based phase recovery process assumes that the object is parallel to the image sensor for all the measurement heights/planes. In practice, however, there usually exists some tilting angle between the object and the image sensor planes, which causes the reconstructions to be aberrated and defocused. A digital tilt correction algorithm^1^ was added to our multi-height based iterative phase recovery process to correct for this artifact^44^,^45^. This rotational field transformation based approach provides a numerical method to calculate the complex optical wavefront on a tilted plane, given the knowledge of the wavefront on the original plane and the angle between the two planes. In the multi-height based iterative phase recovery routine, instead of directly averaging the amplitude guess with the measurement, the guess of the optical wavefront is first rotated to a plane that is parallel to the image sensor before the updating (i.e., averaging) of field amplitudes takes place. After the amplitude updating, the optical wave is then rotated back to the plane parallel to the object. This algorithm requires the knowledge of the tilting angle between the object and the image sensor planes. The tilting angle is calculated by auto-focusing on different regions of the object inside the lens-free field-of-view to obtain the vertical distances at different locations, and a plane is fitted to these points to accurately measure the tilting angle of the object with respect to the sensor plane^1^.

## Results

### Color calibration results of lens-based mobile and benchtop microscopes

We first calibrated the color imaging performance of the two lens-based microscopy devices used in this manuscript – the portable mobile-phone-based microscope built from a smart-phone and the benchtop Olympus BX51 microscope. For the benchtop microscope, the color calibration is mainly targeted for the microscope camera, as the rest of the microscope optics is manufactured to give accurate color reproduction with natural daylight illumination^46^ (color temperature 5500 K); the chromatic aberration is also well corrected. For the mobile-phone based microscope, however, the illumination, external and internal lens modules and the image sensor all contribute to the inaccuracy of its color reproduction, and therefore we calibrated the whole imaging system as a black box.

The custom-fabricated color checkers (Fig. 4(a,c)) and the measured ground truth colors using lens-free hyperspectral imaging (Fig. 4(b,d)) were used to calibrate the mobile-phone microscope and the benchtop microscope following a four-step color calibration procedure as detailed in the Methods Section. Before this calibration (Fig. 4(e,i)), the captured colors were evidently distorted compared to the ground truth (upper-left corner of each patch), which can also be seen in the CIE 1931 chromaticity diagram^25^ (x-y plot) shown in Fig. 4(f,j). The color images taken by our mobile-phone microscope had large amounts of desaturation, which can be easily seen visually, but also is reflected from the x-y plots where the data points aggregate around the center. This is due to the fact that we used the demosaiced raw image directly without saturation enhancement and white-balancing steps that a mobile-phone would normally do, to keep the original signal values and the linearity of pixels. After our digital calibration (Fig. 4(g,k)), for almost all the color patches the upper-left corners merge with the rest, indicating agreement to the ground truth colors. In the x-y plots after calibration (see Fig. 4(h,l)), the calibrated image data points also reside very close to the ground-truth coordinates. Table 1 further quantifies the performance of our color calibration process using the CIE-94 color distance. *After calibration*, *the mean color distances are reduced by a factor of* ~*6 for the mobile-phone based microscope and ~3 for the benchtop microscope*.

**Figure 5:**
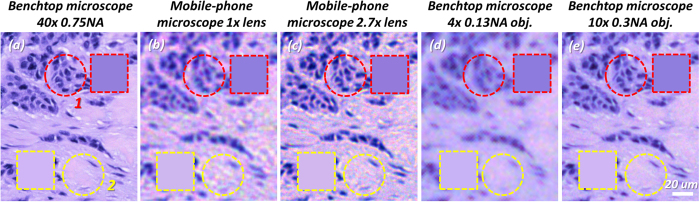
Comparison of color reproduction results of a color-calibrated benchtop microscope and a mobile-phone based microscope by imaging an H&E stained breast cancer tissue section. The colors of the color-calibrated lens-based microscope images are compared against each other to verify the accuracy of color calibration. Even though the resolution and noise levels are much different among different imaging devices and magnifications, the overall colors of all the images are very close to each other. To quantify this agreement, two sub-regions were chosen to calculate the mean RGB values. Sub-region 1 (red dashed circle) mainly consists of cell nuclei, thus the average color of sub-region 1 is a purplish blue (see the patch enclosed by red dashed square). Sub-region 2 (yellow dashed circle) mainly consists of stroma, thus the average color of sub-region 2 is pink (see the patch enclosed by yellow dashed square). The average colors of these two sub-regions and their comparison are summarized in Table 2.

The success of this color calibration process can also be demonstrated by imaging 4-μm thick formalin-fixed paraffin-embedded (FFPE) hematoxylin and eosin (H&E) stained breast cancer tissue sections as illustrated in Fig. 5. For these experiments, we used existing and anonymous specimen, where any subject related information cannot be retrieved. As the tissue is stained with H&E, the image mainly consists of pink and purplish blue colors. The pink-color areas are mostly stroma, whereas the purplish blue colors are mostly cell nuclei. Color accuracy in clinical imaging of such samples is extremely important as color artifacts can be mistaken for other components of tissue and even abnormal signatures, leading to misdiagnosis^47^. Through visual comparison, in Fig. 5 one can see that despite the resolution and noise differences among different microscope and lens combinations, the overall color reproduction in each case is very close to the rest. To quantify the consistency among these different images, we chose two circular sub-regions of 40 μm diameter to calculate the average colors and color distances. Sub-region 1 (red dashed circle) is mainly made up of nuclei (purplish blue), and sub-region 2 (yellow dashed circle) is mainly made up of stroma (pink). The mean values of R, G and B for each sub-region were calculated to compute the color distances of each image against a reference, which was chosen as the color calibrated benchtop microscope with a 40 × 0.75 NA objective lens (Fig. 5(a)). These results are summarized in Table 2: since all the CIE-94 color distances are below 1.5, the differences are barely visible, providing a very good color agreement across various imaging devices and configurations. This cross-validation also shows that our color calibration works very well with the colors of interest in an H&E-stained tissue sample. Even better color calibration outcome could in general be achieved by increasing the sampling in the color space, coupled with adopting other color calibration algorithms such as a look-up table (LUT)^33^ or a neural network^29^.

### Color imaging of tissue sections using lens-free and lens-based image fusion

**Figure 6:**
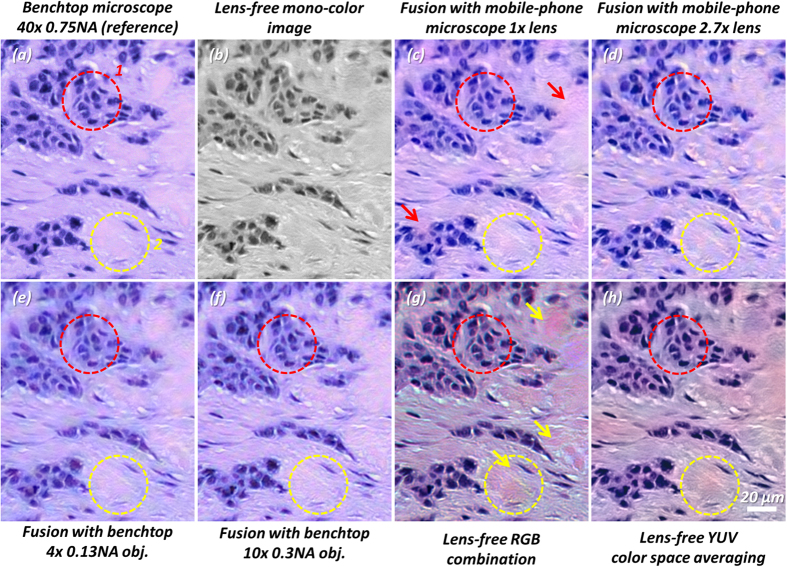
Imaging results of an H&E stained tissue section using different colorization techniques and devices. (**a**) 40 × 0.75 NA benchtop microscope image *after* color calibration. This is used as our reference image to evaluate the color performance and resolution of other techniques. (**b**) The lens-free mono-color intensity image that is used in the image fusion process. (**c**,**d**) Image fusion results of the lens-free image with mobile-phone microscope images using 1×- and 2.7×-magnification, respectively. Due to the relatively low resolution of the 1× magnification geometry, we used a deeper level of wavelet decomposition (N = 6) than the 2.7× magnification geometry (N = 5). As a result, compared to (**d**), there exists a stronger color fluctuation effect in (**c**) as pointed out by the red arrows. (**e**,**f**) Image fusion results of a lens-free image with benchtop microscope images captured using 4 × 0.13 NA and 10 × 0.3 NA objective lenses, respectively. As can be seen in (**e**), there exists some color leakage due to the low-resolution of the 4×-objective image, manifested by the blue color of the nuclei diffusing into the pink-colored stroma region. This effect is much less visible in the fusion result with the 10×-objective image in (**f**). (**g**) Colorization using the direct RGB combination method^2^. Color artifacts are especially evident in the connective tissue regions that show a more reddish color instead of pink (see yellow arrows). (**h**) Colorization using the YUV color space averaging technique^1^,^3^,^11^. Similar to (**g**), the color shows strong distortion compared to the reference image in (**a**). Moreover, it loses the sharpness of color compared to (**g**). The same sub-regions as in Fig. 5 (red and yellow dashed circles), in addition to the entire image, were used in Table 3 to quantify the color performances of these different techniques and imaging devices/configurations.

After demonstrating that our color-calibrated lens-based microscopes generate accurate color reproduction that is consistent across devices and imaging configurations, next we will illustrate the capabilities of DCFM for achieving high resolution and accurate color reproduction over a wide FOV using breast cancer tissue sections. For this aim, we will compare the color imaging results of lens-free microscopy fused with various low-magnification lens-based microscope combinations (i.e., mobile-phone 1×, mobile-phone 2.7×, benchtop 4×, benchtop 10×) against some of the previously used colorization techniques including: (1) lens-free RGB combination based colorization^2^, (2) lens-free YUV color space averaging based colorization^1^,^3^,^11^,^35^,^48^, and (3) a color-calibrated benchtop microscope with a 40 × 0.75 NA objective lens, which is used as our reference. Fig. 6(a) shows the same region of interest as in Fig. 5 captured by the color-calibrated benchtop microscope with a 40 × 0.75 NA objective lens; Fig. 6(b) shows the lens-free single-wavelength intensity reconstruction image that is used as an input to our image fusion approach; and Fig. 6(c–f) show the results of our image fusion method with different microscope-lens combinations. The color reproduction of our DCFM technique, using lower magnification lenses of a benchtop microscope as well as a mobile-phone-based cost-effective microscope, agrees very well with the reference image, yielding almost indiscernible differences. Across different lens-free images that are fused with the mobile-phone based microscope images, color fluctuation artifacts exist in the 1× magnification results (Fig. 6(c), red arrows) showing some pinkish color patches that are not seen in the reference image (Fig. 6(a)). This artifact arises due to the fact that the 1× mobile-phone microscope has relatively low resolution, thus we needed to use a deeper wavelet-decomposition level (N = 6), which couples some of the low-resolution modulation of the lens-free image into the final color representation. On the other hand, Fig. 6(d) is free of such artifacts due to the higher resolution of the 2.7× magnification geometry of the mobile-phone microscope and the shallower wavelet-decomposition level (N = 5) that we used. In the fusion result using the benchtop microscope with a 4 × 0.13 NA objective lens (Fig. 6(e)), one can also notice that the purplish blue color of the nuclei partially diffuses into the surrounding tissue, as a result of the low resolution of the 4× objective lens. In comparison, the same color diffusion effect/artifact is not found in our image fusion results using the benchtop microscope image taken with a 10 × 0.3 NA objective lens for the same reasons detailed above (Fig. 6(f)). To expand our comparison, Fig. 6(g) shows the colorization result of the RGB-combination method^1^,^2^,^3^,^11^ which utilized holographic reconstructions at three wavelengths (B: 471 nm, G: 532 nm, R: 633 nm) using purely the lens-free on-chip microscope. In this case, the stroma is more reddish than the reference image, and the nuclei appear more purplish. More importantly, at certain locations (labeled by the yellow arrows) color artifacts are relatively strong, showing reddish and yellowish colors. As another comparison, Fig. 6(h) shows the result obtained using the lens-free YUV color space averaging method^1^,^3^,^11^ using illumination wavelengths of 471 nm, 532 nm and 633 nm. As spatial blurring is done to the chroma components in this technique, the color is not only distorted similar to Fig. 6(g), but also partially blurred.

To better quantify and summarize the above described results and image comparisons across different devices and colorization techniques, we also calculated the color distances from the reference image in Table 3, similar to the presentation in Table 2. Sub-regions 1 and 2 were the same regions of interest as defined in Fig. 5. We also added a new region that is shown in Fig. 6. Furthermore, we added another color-distance measure defined as the mean CIE-94 color distance on a pixel-by-pixel comparison basis. This new measure is expected to be larger than the color distance of the region-average RGB, as the differences in resolution and spatial details are also included in its calculation. For all the image fusion results, the pixel-by-pixel mean color distances from the reference image mostly fall below 3.5 and are on a similar level, except for the fusion with the benchtop microscope image with a 4 × 0.13 NA objective lens that has a larger color error for sub-region 1. This, again, can be explained by the diffusion of the local image colors into their vicinity as a result of the low resolution of the 4× image used for fusion, and this causes the details of Fig. 6(e) to deviate from the reference image. Interestingly, the pixel-by-pixel average color error values of the fusion results using the mobile-phone microscope are very close to each other. Meanwhile, the pixel-by-pixel mean color distances of the lens-free RGB combination and lens-free YUV color space averaging techniques are almost twice larger than the image fusion results, implying worse color reproduction.

**Figure 7:**
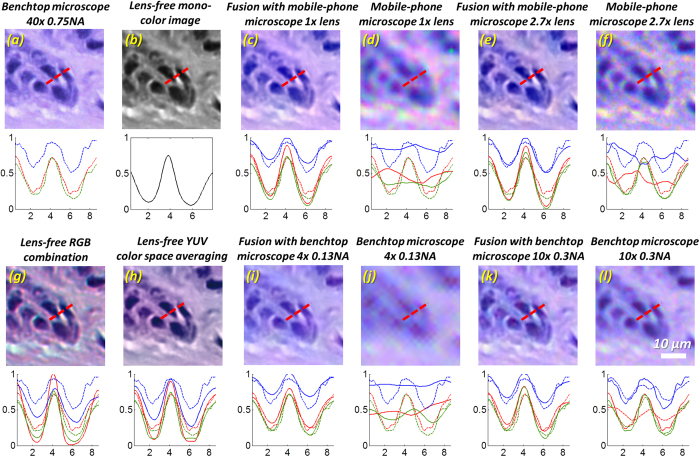
Comparison of image resolution among different colorization techniques. A line profile across two closely located nuclei is measured along the same position of each image (red dashed line) and plotted below each image. The line profile of the lens-free grayscale image is drawn in black solid line (**b**) lower panel. For all the other images, the line profiles are drawn in red, green and blue lines representing the R, G and B components, where the solid lines represent the line profile of the current image, and the dashed lines representing the line profile of the reference 40 × 0.75 NA microscope image. The line profiles of the fused images (**c**,**e**,**i**,**k**) better resemble the reference image preserving the main spatial features. The lens-free RGB and lens-free YUV colorization methods (**g**,**h**) resolve the two dips in the curves representing the two nuclei, but they both exhibit color distortions.

As stated previously, besides benefiting from accurate color reproduction of lens-based color imaging devices, the presented DCFM method also benefits from lens-free microscopy by achieving high resolution across a large FOV. In Fig. 7 we zoom into a smaller region of the tissue sample to evaluate and compare the preservation of sharp features at cell boundaries, where we also present a one-dimensional line profile across two nuclei separated by a narrow gap. In Fig. 7(a), our reference image that is captured by a color calibrated benchtop microscope using a 40 × 0.75 NA objective lens is shown. In Fig 7(b), the same region of the lens-free intensity image is shown in grayscale. For all the other panels, below each image, the solid curves (red, green and blue, corresponding to the R, G and B channels, respectively) show the line profiles of the current image, and the dashed curves show the line profiles of the reference image, presented for comparison. As shown in Fig. 7(d,f,j,l), the images acquired using the low-magnification benchtop microscope and the mobile-phone microscope are low-resolution, with the spatial features (e.g., the line profile dips between the nuclei) either completely lost (Fig. 7(d,j)) or significantly reduced in contrast (Fig. 7(f,l)). Our DCFM method, on the other hand, clearly shows the spatial features of the nuclei, and the line profiles agree very well with the reference image. The line profiles of the lens-free RGB combination and lens-free YUV color space averaging methods exhibit similar spatial resolution as the lens-free grayscale image, but the curves deviate from the reference image due to inaccurate color representation, further emphasizing the advantages of the presented image fusion based colorization approach.

## Discussion

We have shown in the Results section that the DCFM technique can achieve high resolution and color fidelity simultaneously. However, another important advantage lies in its FOV. Lens-free on-chip microscopy has a FOV that is equivalent to the active area of the imager chip, which is ~20.5 mm^2^ in the experiments that we report in this manuscript. Nevertheless, this is not the case with lens-based imaging systems; for example a 40× objective lens typically has a FOV of ~0.1 mm^2^. In our image fusion based colorization method, to better make use of the large FOV provided by lens-free on-chip microscopy, we utilized low-magnification and low-NA lens-based microscopes to provide color-calibrated large FOV images to be merged with the reconstructed high-resolution lens-free images. To further expand on this, Table 4 summarizes the imaging FOVs of different microscope-lens combinations that we used in this work. For example, the mobile-phone microscope with a 1× magnification system has a FOV of 3.9 mm^2^. To match the FOV of our lens-free microscope used in this work, one needs to stitch >5 images together. For the benchtop microscope, on the other hand, one needs to stitch approximately 2 images for the 4× objective lens and >11 images for the 10× objective lens. Capturing the images of these different FOVs can be achieved by e.g., manually scanning the sample, and the digital stitching of different images can be automated through image registration.

Next, we would like to discuss the depth-of-field (DOF) of DCFM. It is widely known that holographic imaging has an extended DOF compared to traditional incoherent light microscopy thanks to the knowledge of the complex optical wave. By digitally propagating the object wave to different planes, the features at different depths can be brought into focus. This is an important capability especially for imaging samples that have 3D morphology over the sample FOV^49^. An important question is whether this freedom of 3D digital focusing could still be useful if the lens-free image were to be fused with a lens-based incoherent microscope image. Fortunately, since we are using a low-magnification and low-NA lens-based imaging system, the DOF of the lens-based image is also quite large. Assuming an average wavelength of 550 nm in air, a 4 × 0.13 NA microscope objective has a DOF of ~32 μm. As another example, our mobile-phone microscope with 2.7× magnification has an NA of approximately 0.25, with a DOF of ~9 μm. As a result of these, we can conclude that a color image that is fused using our technique can be digitally focused over a relatively large depth range over the sample volume by simply refocusing the lens-free image before the image fusion step.

We should also note a limitation of the presented DCFM method: the resolution of its color (chroma) component is actually inferior to the resolution of its brightness component, as the source of the color information is a low-NA lens-based microscope. The success of our strategy relies on the widely accepted assumption that in most scenarios, the chroma (color) does not vary as fast as the brightness. This assumption is also used in high-quality de-bayering of raw color images^50^. For example, in pathology the most important spatial details are usually at the cell boundaries or sub-cellular textures which are mostly brightness variations^3^, thus the lower resolution of the color component would be acceptable.

Another issue that is worth discussing is the optical aberrations across the entire imaging FOV, which is especially significant for mobile-phone-based cost-effective microscope images. The external lenses used in mobile-phone microscopes are in general low-cost compound lens modules, also taken from mobile-phone cameras in many cases, which are not optimized for microscopy purposes. As a result, the image is not as sharp at the edges of the FOV as it is at the center, the focal distances for R, G, and B channels are slightly different, and in addition to these, there exists a dislocation among the R, G, and B channels. Besides using higher-quality lenses, alternative approaches to mitigate aberrations include capturing more images that focus on R, G and B channels individually and use only the center of the FOV where the image is the sharpest. These approaches will either relatively raise the cost of the system or increase the image acquisition time, which might not be acceptable in certain applications. In this work, we only digitally corrected for the displacement among the R, G, B channels using an image registration algorithm and neglected the other sources of aberrations. Although these remaining aberrations due to poor optical components/lenses inevitably affect our results, their impact on spatial resolution is not as critical since in our image fusion approach we do not rely on the lens-based image for resolution.

In terms of further simplification and cost-reduction of our proof-of-concept DCFM set-up demonstrated in this work, we would like to emphasize the following points. First, instead of the benchtop super-continuum source used in our experiments, low-cost LEDs that are butt-coupled to multi-mode fibers (with e.g., 0.05 mm core size) can be used as illumination sources without the use of any alignment optics or beam focusing^3^,^4^,^7^,^21^. Due to the relatively wide spectral bandwidths of typical LEDs, thin-film color filters can also be used to improve the temporal coherence of the illumination. Second, instead of using an automated positioning stage to achieve sample movement in the x-y plane (for PSR) and the z-direction (for multi-height based phase recovery), an array of LEDs can be used to achieve PSR^3^,^7^,^21^, and custom-made cost-effective manual z-shift stages can be used to achieve the relative shifts in the z-direction to be used for multi-height phase recovery^3^,^7^. We can utilize these cost-effective and simpler solutions because of the robustness of the PSR and multi-height based phase recovery algorithms. For example, the PSR algorithm contains a shift-estimation module to precisely calculate the sub-pixel shifts between the low-resolution holograms, and the super-resolved hologram is obtained from these multiple sub-pixel shifted holograms by solving a non-linear optimization problem^3^,^7^. Thus, the x-y shifts do not need to be on a regular grid. Similarly, the sample-to-sensor distances are precisely estimated through an auto-focus algorithm, and therefore the z-shifts need not be known *a priori*^1^,^3^,^6^,^7^.

Finally, for this DCFM technique to be easily used by non-experts, modularization of the image reconstruction and color fusion algorithms and simplification of the user interface will be necessary. The control programs and image processing algorithms need to be well developed such that the technical details are concealed from the users, leaving them with a selection of a few parameters through a user-friendly and simple graphical interface.

## Conclusion

We reported a novel microscopy technique (DCFM) that can achieve high color fidelity and high resolution across a wide FOV by combining a holographic image acquired at a single wavelength with a color-calibrated and low-resolution lens-based image using a wavelet-based image fusion algorithm. This method combines the wide FOV and high resolution advantages of lens-free holographic microscopy with accurate color reproduction of a color-calibrated lens-based bright-field microscope, generating images that match the chromatic perception of human vision. Using this method we successfully imaged tissue samples, and demonstrated that by combining a lens-free microscope with a low-cost mobile-phone-based microscope, accurate color images of specimen can be obtained, coming very close to the images of a high-NA and color-calibrated benchtop microscope. This method might present a promising solution for telepathology applications in resource limited environments, where digital whole-slide scanners are not available.

## Additional Information

**How to cite this article**: Zhang, Y. *et al*. Color calibration and fusion of lens-free and mobile-phone microscopy images for high-resolution and accurate color reproduction. *Sci. Rep.* **6**, 27811; doi: 10.1038/srep27811 (2016).

## Supplementary Material

Supplementary Information

## Figures and Tables

**Figure 1:**
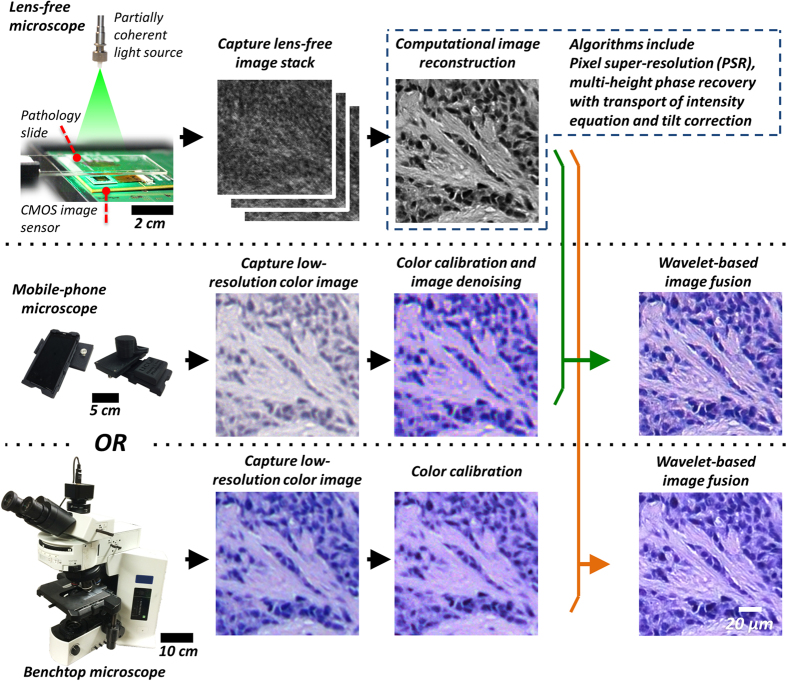
Optical setup and imaging flow chart. Top row: the lens-free microscope (single-wavelength illumination) captures multiple x-y-z shifted holographic images, and uses image reconstruction algorithms to recover a high-resolution grayscale image of the object over a large FOV of e.g., ~20 mm^2^. Middle row: a mobile-phone microscope is used to capture relatively lower-resolution images of the corresponding regions of the same sample. This color image from the mobile-phone microscope is combined with the high-resolution lens-free image using a wavelet-based image fusion algorithm, achieving accurate color representation with high resolution. Bottom row: similar to the middle row, except this time for a benchtop microscope with a low-numerical-aperture (NA) objective lens.

**Table 1:** Color calibration results of the mobile-phone based microscope and the benchtop microscope.

Microscope/objective	CIE-94 color distance before color calibration			CIE-94 color distance after color calibration		
	mean	max	min	mean	max	min
Mobile-phone microscope 1× lens	12.43	22.82	0.38	2.02	7.07	0.18
Mobile-phone microscope 2.7× lens	13.58	26.35	0.46	2.36	6.99	0.60
Benchtop microscope 4 × 0.13 NA obj.	6.24	15.35	0.85	2.31	8.87	0.36
Benchtop microscope 10 × 0.3 NA obj.	6.65	16.03	0.79	2.43	9.07	0.42
Benchtop microscope 40 × 0.75 NA obj.	6.33	15.85	0.87	2.38	7.65	0.37

For the mobile-phone microscope, the mean CIE-94 color distances from the ground truth colors were >12 before calibration, and reduced to <2.5 after calibration. For the benchtop microscope, the mean CIE-94 color distances from the ground truth colors were >6 before calibration, and reduced to <2.5 after calibration.

**Table 2:** Calibrated average colors of sub-regions 1 and 2 shown in Fig. 5 imaged by different devices.

Microscope/objective	Sub-region 1 (nucleus)		Sub-region 2 (stroma)	
	Region mean R,G,B	Color distance from benchtop 40×	Region mean R,G,B	Color distance from benchtop 40×
Benchtop microscope 40 × 0.75 NA obj.	0.58, 0.50, 0.86	0	0.82, 0.71, 0.96	0
Mobile-phone microscope 1× lens	0.58, 0.48, 0.86	1.21	0.82, 0.72, 0.93	1.36
Mobile-phone microscope 2.7× lens	0.56, 0.50, 0.87	0.93	0.80, 0.72, 0.93	1.46
Benchtop microscope 4 × 0.13 NA obj.	0.55, 0.49, 0.87	1.27	0.78, 0.70, 0.96	1.24
Benchtop microscope 10 × 0.3 NA obj.	0.56, 0.49, 0.87	0.79	0.81, 0.71, 0.96	0.42

As shown in this table, all the color distances are below 1.5, verifying the effectiveness of the presented color calibration method.

**Table 3:** Quantification of color distances.

Imaging and colorization technique	Entire image		Sub-region 1 (nucleus)		Sub-region 2 (stroma)	
	Pixel-by-pixel	Region average	Pixel-by-pixel	Region average	Pixel-by-pixel	Region average
Fusion with mobile-phone microscope, 1×	3.31	0.52	3.36	0.97	2.83	1.12
Fusion with mobile-phone microscope, 2.7×	3.26	0.42	3.26	0.86	2.69	0.73
Fusion with benchtop microscope, 4 × 0.13 NA	3.51	1.13	4.18	1.27	2.69	1.24
Fusion with benchtop microscope, 10 × 0.3 NA	3.03	0.46	3.34	0.79	2.47	0.42
Lens-free RGB combination	5.92	2.81	6.31	2.50	5.09	3.15
Lens-free YUV color space averaging	8.76	3.64	10.44	2.65	6.86	5.44

Two different CIE-94 mean color distance measures were calculated. (a) CIE-94 color distance from the reference 40× color-calibrated benchtop microscope image was calculated for each individual pixel and averaged. (b) R, G, and B values of the region of interest were averaged, and the CIE-94 color distance of the averaged RGB was calculated from the reference image (also RGB-averaged in the corresponding region). Our image fusion results show significant improvements in reduction of color distances.

**Table 4:** Comparison of FOVs and effective pixel sizes of different microscopic imaging systems discussed in this paper.

Microscope/objective	Effective pixel size (μm)	FOV (mm^2^)	# of tiles to match the lens-free FOV
Lens-free microscope	0.37	20.5	1
Mobile-phone microscope, 1×	1.14	3.9	>5
Mobile-phone microscope, 2.7×	0.46	2.1	~10
Benchtop microscope 4 × 0.13 NA obj.	1.52	11.6	~2
Benchtop microscope 10 × 0.3 NA obj.	0.60	1.8	>11
Benchtop microscope 40 × 0.75 NA obj.	0.15	0.1	>200
