# Supplementary material for: Color calibration and fusion of lens-free and mobile-phone microscopy images for high-resolution and accurate color reproduction
Source: Sci Rep. 2016 Jun 10;6:27811. doi: 10.1038/srep27811 (PMC4901265; doi:10.1038/srep27811)
Supplement: Supplementary Information [file srep27811-s1.pdf]

Supplementary information for

Color calibration and fusion of lens-free and mobile-phone microscopy images for high-resolution and accurate color reproduction

Authors: Yibo Zhang[1,2,3], Yichen Wu[1,2,3], Yun Zhang[1], and Aydogan Ozcan[1,2,3,4,*]

Affiliations:

[1] Electrical Engineering Department, University of California, Los Angeles, CA, 90095, USA.

[2] Bioengineering Department, University of California, Los Angeles, CA, 90095, USA.

[3] California NanoSystems Institute (CNSI), University of California, Los Angeles, CA, 90095, USA.

[4] Department of Surgery, David Geffen School of Medicine, University of California, Los Angeles, CA, 90095, USA.

*Correspondence: Prof. Aydogan Ozcan

E-mail: ozcan@ucla.edu

Address: 420 Westwood Plaza, Engr. IV 68-119, UCLA, Los Angeles, CA 90095, USA

Tel: +1(310)825-0915

Fax: +1(310)206-4685

Authors' email addresses:

Yibo Zhang	zybmax@ucla.edu
Yichen Wu	wuyichen@ucla.edu
Yun Zhang	yyyunzhang@ucla.edu
Aydogan Ozcan	ozcan@ucla.edu

Supplementary Figures

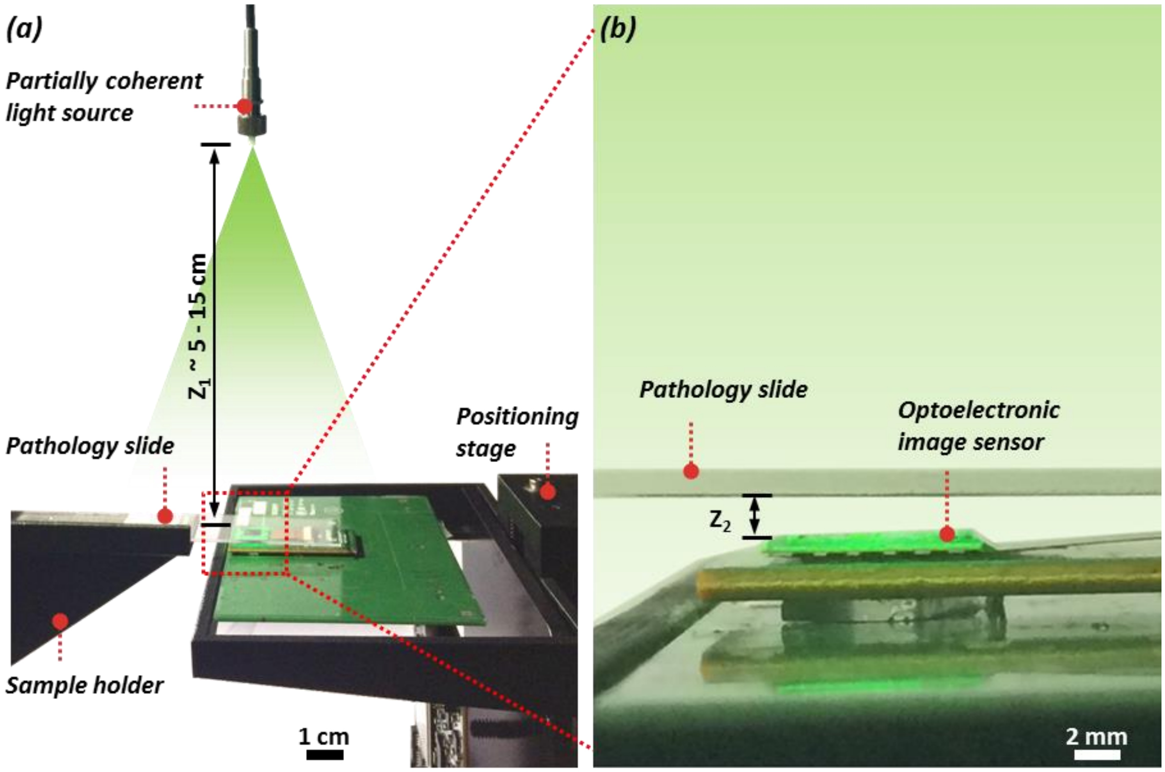

Supplementary Figure S1: (a) Lens-free on-chip microscopy setup. (b) Zoom-in view of the relative position of the sample and the image sensor chip. The image sensor chip is placed below the sample with a typical z_2 distance of < 1 mm. The z_2 distance in this figure is enlarged for ease of visualization.

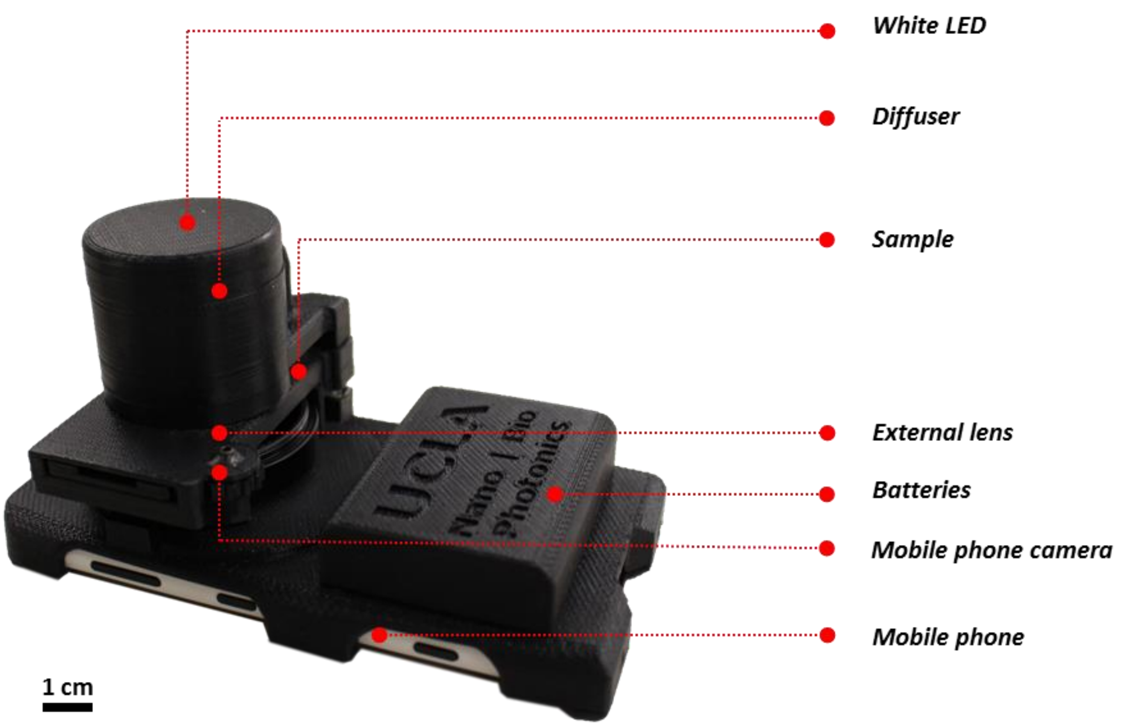

Supplementary Figure S2: Mobile-phone based microscope optical design. The light from a white LED passes through a diffuser to illuminate the sample (e.g., a pathology slide). The sample is mounted on a custom-fabricated x-y-z translation stage. An external lens is placed on top of the mobile phone camera to provide magnification. When operated, the mobile-phone microscope is held in a vertical position.
